# Unilateral facial palsy with hemiplegia and positive anti-GT1a antibody: a case report and literature review

**DOI:** 10.3389/fimmu.2026.1793550

**Published:** 2026-04-22

**Authors:** Fangfang Wang, Jing Miao

**Affiliations:** Department of Neurology, The First Hospital of Jilin University, Changchun, China

**Keywords:** GBS, GT1a, Guillain-Barré syndrome, unilateral facial palsy, unilateral limb weakness

## Abstract

**Background:**

Guillain-Barré syndrome (GBS) is an autoimmune-mediated disorder characterized by an acute onset of symmetrical flaccid limb weakness. However, cases of persistent unilateral limb weakness and facial palsy with anti-GT1a antibody positivity have been rarely reported. Here, we describe this case to highlight its clinical importance.

**Case:**

A 60-year-old man presented with right shoulder and upper back pain, followed by right-sided limb weakness, peripheral facial paralysis, distal sensory impairment, and dyspnea. Symptoms were preceded by upper respiratory infection. Cerebrospinal fluid showed albuminocytological dissociation. Electromyography reported peripheral nerve damage in the extremities. Serum anti-GT1a IgG was positive. After a five-day course of intravenous immunoglobulin, he showed marked recovery in motor, sensory, and respiratory function. At six-month follow-up, his symptoms except dyspnea basically returned to normal.

**Conclusion:**

This case suggests a potential distinct GBS phenotype featuring unilateral limb weakness, unilateral facial palsy, and anti-GT1a antibody positivity, with a good response to IVIG. These findings expand the clinical spectrum of GBS and emphasize recognizing unilateral presentations to prevent misdiagnosis.

## 1 Introduction

Guillain-Barré syndrome (GBS) is an acute autoimmune-mediated polyspinal radiculopathy, a concept first reported by Guillain (1). Patients typically have a history of prior infection before the onset of disease. The clinical features include symmetrical flaccid limb weakness, diminished or absent deep tendon reflexes, and sensory abnormalities (2). Notably, symmetry often serves as a primary diagnostic criterion for classical subtypes of GBS. Besides the major classical GBS subtype (such as acute motor axonal neuropathy, AMAN), it also encompasses various variant types of GBS characterized by local involvement. According to epidemiological statistics from North America and Europe, the median overall incidence rate of GBS is 1.11 cases per 100,000 person-years, with an exponential increase with age (3). However, the incidence of GBS with unilateral limb weakness may be less than 1% (4). In recent years, the variant forms of GBS have attracted increasing attention. For instance, a prospective study of 490 GBS patients by van den Berg et al. demonstrated that most patients with paraplegic GBS exhibit diminished or absent upper limb tendon reflexes, sensory disturbances, or abnormal electromyography, despite preserved upper limb muscle strength (5). This finding suggests that limited limb weakness is merely a superficial manifestation of diffuse peripheral nerve involvement. Additionally, Wakerley and Yuki reported up to 20% (3/15) of patients with asymmetry cranial nerve involvement in their cranial polyneuritis case series, a recognized feature of cranial nerve variants in GBS (6). This study indicates that the clinical feature of asymmetry alone cannot be used as a criterion to exclude a diagnosis of GBS. To date, only Maharjan et al. have reported a single case in 2023 of a GBS patient who presented with unilateral motor weakness at onset and subsequently progressed to bilateral limb weakness (7). Herein, we report a novel case of GBS with right-sided limb weakness, right facial palsy and positive anti-GT1a antibody. This case and the previously reported case (7) suggest that the unique presentation of unilateral facial paralysis and limb weakness may constitute a rare phenotype of GBS rather than an accidental occurrence. Through a detailed comparison of these two cases and in combination with literature reviews, we aim to expand the understanding of the GBS clinical spectrum.

## 2 Case report

A 60-year-old male patient was admitted to our hospital with a 10-day history of right shoulder and upper back pain. Seven days before hospitalization, he received treatment with cephalosporin antibiotics and methylprednisolone at a local hospital but experienced no improvement. Three days prior to admission, his symptoms worsened and weakness of the right-sided limbs developed, mainly manifested as an inability to raise the right upper limb or squat down, unsteady walking, and difficulty maintaining a standing posture. In addition, he presented with absence of the right forehead wrinkles and deviation of the right corner of his mouth. Dyspnea occurred both on exertion and at rest. He was subsequently treated with an intravenous infusion of nemonoxacin malate in our emergency department; however, no significant improvement was observed. Ten days before the onset of symptoms, he experienced coughing without fever. The patient reported no dysphagia or headache during the course of the illness. Since the onset of the illness, the patient has had normal sleep, appetite, bowel movements, and urination. No significant changes in body weight have been observed recently.

Physical examination showed right peripheral facial palsy. For muscle strength assessment, the Medical Research Council (MRC) scale grades muscle strength from 0/5 (no contraction) to 5/5 (normal). In this patient, strength testing using this scale revealed that the right upper limb proximal muscles were graded 2/5, and distal muscles 4/5; the right lower limb proximal muscles were graded 3/5, and distal muscles 3/5; all left limb muscles were graded 5/5. The MRC sum score was 47/60. Muscle tone was normal. Deep tendon reflexes were absent in all limbs. Glove-and-stocking distribution hypoesthesia to pinprick was noted in the distal portions of all four extremities, with intact vibration and proprioception. The pathological reflexes (such as Babinski, Chaddock, Hoffman) were negative. Electromyography (EMG) was performed 16 days after the onset of illness. Conduction block (>30% drop in CMAP amplitude) was present in the left median and bilateral peroneal nerves, indicating demyelinating motor neuropathy. Other findings included prolonged F-wave latencies, reduced motor conduction velocities, and delayed distal latencies in multiple nerves. Sensory nerve action potentials were reduced, confirming sensory fiber involvement. Active denervation was observed on needle EMG (**Table 1**).

**Table 1:** Comparison of electromyography between the left and right limbs of this patient.

Motor nerve conduction	Left						Right (symptomatic)					
	DML	CMAP		MCV		MFL	DML	CMAP		MCV		MFL
	(ms)	(mV)		(m/s)		(ms)	(ms)	(mV)		(m/s)		(ms)
Ulnar												
Wrist – ADM	**3.23** *(<3.0)*	10.4*(>5.0)*				**37.0** *(<28.0)*	2.92*(<3.0)*	11.4*(>5.0)*				**32.3** *(<28.0)*
Below elbow		9.2*(>5.0)*		**46.6** *(>50.0)*				9.6*(>5.0)*		**43.7** *(>50.0)*		
Above elbow		9.3*(>5.0)*		**47.4** *(>50.0)*				9.0*(>5.0)*		**40.7** *(>50.0)*		
Median												
Wrist – APB	**4.02** *(<4.0)*	10.4*(>6.0)*				**38.4** *(<28.0)*	**4.54** *(<4.0)*	6.8*(>6.0)*				**32.8** *(<28.0)*
Elbow		7.1*(>6.0)		**39.2** *(>50.0)*				**5.4** *(>6.0)*		**37.4** *(>50.0)*		
Tibial												
Ankle – AH	4.17*(<4.5)*	7.5*(>5.0)*				**68.3** *(<53.0)*	**4.56** *(<4.5)*	10.2*(>5.0)*				**64.8** *(<53.0)*
Popliteal fossa		6.3*(>5.0)*		**34.6** *(>40.0)*				6.5*(>5.0)*		**33.8** *(>40.0)*		
Peroneal												
Ankle – EDB	3.46*(<5.0)*	4.3*(>2.5)*					4.42*(<5.0)*	3.4*(>2.5)*				
Below knee		2.2* *(>2.5)*		**30.2** *(>40.0)*				2.1* *(>2.5)*		**32.0** *(>40.0)*		
Above knee		**2.0** *(>2.5)*		**32.0** *(>40.0)*				**2.3** *(>2.5)*		**36.4** *(>40.0)*		
Sensory nerve conduction	SAP			SCV			SAP			SCV		
	(µV)			(m/s)			(µV)			(m/s)		
Ulnar												
Wrist - Finger V	**7.6** *(>10.0)*			**41.1** *(>50.0)*			**2.4** *(>10.0)*			**44.7** *(>50.0)*		
Radial												
Wrist - Finger I	17.0*(>10.0)*			**49.0** *(>50.0)*			11.8*(>10.0)*			**46.3** *(>50.0)*		
Median												
Wrist - Finger II	**4.1** *(>10.0)*			**44.3** *(>50.0)*			**7.4** *(>10.0)*			**42.4** *(>50.0)*		
Wrist - Finger III	**4.2** *(>10.0)*			**40.3** *(>50.0)*			**3.3** *(>10.0)*			**41.0** *(>50.0)*		
Superficial peroneal												
Calf – Foot	8.7*(>5.0)*			**39.0** *(>40.0)*			**3.2** *(>5.0)*			40.5*(>40.0)*		
Sural												
Calf – Ankle	13.2*(>5.0)*			**37.9** *(>40.0)*			5.8*(>5.0)*			**40.0** *(>40.0)*		
Needle EMG	Spontaneous activity				Recruitment pattern		Spontaneous activity				Recruitment pattern	
	Fib		PSW				Fib		PSW			
Infraspinatus	0		0		Normal		**1+**		**1+**		Normal	
Deltoid	NT		NT		NT		**2+**		**2+**		Mildly reduced	
Biceps	NT		NT		NT		0		0		Normal	
ADM	NT		NT		NT		0		0		Normal	
APB	NT		NT		NT		0		0		Normal	
Gastrocnemius	0		0		Normal		0		0		Normal	
Tibialis anterior	0		0		Normal		0		0		Normal	

EMG, Electromyography; ADM, abductor digiti minimi; APB, abductor pollicis brevis; AH, adductor hallucis; EDB, extensor digitorum brevis; DML, distal motor latency; CMAP, compound muscle action potential; MCV, motor conduction velocity; MFL, mean F-Wave latency; SAP, sensory action amplitude; SCV, sensory conduction velocity; ms, milliseconds; mV, millivolt; m/s, meter/second; µV, microvolt; Fib, fibrillation potential; PSW, positive sharp wave; NT, not tested; Spontaneous activity was graded semi-quantitatively on a scale of 0 to 4+, (0 = normal, 1+ = persistent runs in at least two areas, 2+ = moderate number in three or more areas, 3+ = large, 4+ = filling the baseline) (24). Recruitment pattern: normal, mildly reduced, severely reduced, early, poor activation. Abnormal values are in bold. Normal reference ranges are in italics. *Conduction block of more than 30%. Due to patient discomfort during the procedure, not all muscles or examination items were tested.

Cerebrospinal fluid (CSF) analysis revealed albuminocytological dissociation, characterized by an elevated protein level of 2.09 g/L (reference 0.15-0.45 g/L) and a normal cell count of 7 × 10^6^/L (reference 0-8 × 10^6^/L). The CSF immunoglobulin G (IgG) level was elevated at 666.61 mg/L (reference 0–34 mg/L). No abnormalities were found on magnetic resonance imaging (MRI) scans of the head. MRI of the cervical spine showed osteophytes and disc bulging at the C2-C3 and C6-C7 levels, as well as disc protrusion at the C5-C6 level and thickening of the ligamentum flavum at the C4-C6 and C6-C7 levels. The brachial plexus MRI revealed no specific changes. Serum and cerebrospinal fluid analyses were negative for paranodal antibodies (NF155, NF186, CNTN1, CNTN2, and CASPR1), as well as gangliosides (sulfatide, GM1, GM2, GM3, GM4, GD1a, GD1b, GD2, GD3, GT1b, and GQ1b). Only anti-GT1a IgG in the serum was positive.

Ultimately, he was diagnosed with Guillain-Barré syndrome. The patient received a five-day course of intravenous immunoglobulin (IVIG) therapy at a dose of 0.4 grams per kilogram per day. On the fourth day of treatment, his dyspnea at rest improved. Three days after completing IVIG therapy, the distal hypoesthesia to pinprick in the right limbs improved. On the fifth day post-treatment, he reported subjective improvement of a limb weakness, though objective muscle strength remained unchanged. Two months after discharge, his right upper limb weakness, facial paralysis and distal sensory hypoesthesia of all limbs improved. He was able to perform minor daily activities, such as washing and walking up to 200 meters without assistance. However, dyspnea persisted on exertion. Right lower limb weakness had no obvious improvement. Six months after discharge, most symptoms were basically recovered. He could squat, lift a heavy object such as a 10-liter water container, and walk up to one kilometer independently. Only dyspnea was present during intense activity.

## 3 Discussion

This patient presented with acute illness marked by initial radicular pain, rapidly followed by progressive motor weakness affecting the unilateral limbs and facial nerve. While unilateral involvement may initially indicate a central process, the combination of limb areflexia, distal sensory loss, and EMG findings pointed to a peripheral nerve disorder. CSF analysis showed albuminocytological dissociation, and serum anti-GT1a antibody was positive. The above key laboratory results suggest immune-related lesions. Moreover, he showed no response to glucocorticoids and antibiotics but improved after IVIG. Collectively, the patient meets NINDS clinical criteria for GBS (8).

### 3.1 Differential diagnosis

For patients with acute unilateral limb weakness and facial paralysis, a careful differential diagnosis of central and peripheral lesions is imperative.

#### 3.1.1 Central nervous system

Cerebral lesions such as acute ischemic stroke, multiple sclerosis, and progressive multifocal leukoencephalopathy were considered. However, no acute infarct, demyelinating lesion, or white matter abnormality was detected on cranial MRI, and pathological reflexes were absent; therefore, the above diagnoses were ruled out. Spinal cord lesions including acute flaccid myelitis and cervical spondylotic myelopathy were also considered, but the patient had no other upper motor neuron signs, and cervical MRI found no specific lesions, so these conditions could also be ruled out.

#### 3.1.2 Peripheral nervous system

Additionally, radicular and peripheral disorders like cervical radiculopathy and brachial plexus neuritis also require differentiation. Such diseases usually present with sensorimotor dysfunction limited to a single nerve root or unilateral upper limb. The cervical MRI did not show any structural lesions, and the EMG was more consistent with a peripheral neuropathy rather than a plexopathy. Therefore, the above diseases could be excluded.

#### 3.1.3 GBS variant

After excluding the above central and peripheral causes, the diagnosis centers on GBS. In the GBS spectrum, pharyngo-cervical-brachial (PCB) is a focal variant closely associated with anti-GT1a antibodies (9). It features acute bulbar palsy (dysphagia and dysarthria), cervical weakness, and bilateral upper limb weakness. Facial paralysis is often observed, while lower limbs are typically unaffected or only mildly involved. Both this case and PCB share acute onset, facial palsy, and anti-GT1a positivity. However, our patient did not develop bulbar palsy or neck weakness, and the neurological involvement was not symmetrical.

These findings indicate that the case is not a typical PCB variant, but rather an atypical phenotype within the broader spectrum of anti-GT1a positive GBS.

### 3.2 Comparison with a similar case

A previously reported case by Maharjan et al. (7) shared several features with our patient. They both presented with acute right-sided onset facial paralysis, limb weakness, and ipsilateral radicular pain. Besides, they also had similar results on EMG and CSF, and both responded well to IVIG. Despite these similarities, we hope to conduct a comparative analysis to explore important features of this potential phenotype (**Table 2**).

**Table 2:** Clinical characteristics of the two patients.

	Case 1	Case 2
**Demographic characteristics**	60 years old, male	39 years old, male
**Pre-infective**	Cough but not fever	Fever
Clinical manifestations		
Cranial Nerve	Right facial paralysis	Right facial paralysis
Motor and Sensory	***Right limbs weakness***	Right lower limb weakness →***symmetric quadriparesis***
	Right shoulder and back pain	Right lower limb pain
Dyspnea	***Yes***	No
Neurological examinations		
MRC	R UL: 2 (prox.)/4 (dist.)	R UL: 4
	R LL: 3	R LL: 3
	L UL: 5	L UL: 4
	L LL: 5	L LL: 3
Tendon Reflexes	***Absent in all limbs***	Absent at right ankle and knee
Superficial Sensation	***Reduced pinprick sensation in the distal extremities***	Reduced tactile sensation in the right lower limb
Deep Sensation	Yes	Yes
Laboratory investigations		
CSF	Albuminocytological dissociation	Albuminocytological dissociation
EMG	Demyelination	Demyelination
**Imaging**	Head and brachial plexus MRI revealed no abnormal changes;	Head and spinal MRI showed no abnormal findings;
	Cervical spine MRI showed degenerative changes.	Lumbar MRI revealed mild degenerative changes.
**Ganglioside antibodies**	***Serum anti-GT1a IgG***	Not detected/Not reported
**Treatment**	IVIG	IVIG

Case 1, our report; Case 2, Maharjan et al.’s report (7); MRC, Muscle Strength Grading; R UL, Right Upper Limb; R LL, Right Lower Limb; L UL, Left Upper Limb; L LL, Left Lower Limb; (prox.), proximal; (dist.), distal. The bolded and italicized sections are more characteristic differentiating points between the two cases. Both patients met GBS diagnostic criteria. Case 1 had right limb weakness, dyspnea, and anti-GT1a positivity; Case 2 showed pain, areflexia, and reduced tactile sensation of the right lower limb, without respiratory involvement or detectable anti-GT1a antibody. Despite these differences, both had demyelinating features on EMG and responded well to IVIG.

#### 3.2.1 Unilateral limb weakness

Notably, this case diverges fundamentally from the case report by Maharjan et al. in disease progression (7). Our case presents symptoms persistently confined to the right side, while the comparative case (7) eventually progressed to bilateral symmetric involvement. This difference may be attributed to specific ganglioside antibodies. It is reported that anti-GT1a Ab may target GT1a glycolipids enriched in peripheral nerves (such as glossopharyngeal, vagus and facial nerves), the medulla oblongata, cervical-brachial plexus, as well as the trigeminal nucleus and dorsal horn of the spinal cord (6, 9–12). Our case detected anti-GT1a-IgG positivity, potentially limiting the immune attack to the right side. In contrast, the control case (7) had no reported anti-GT1a antibody, allowing unrestrained immune attack to extend to the contralateral side. This comparison shows that unilateral manifestations cannot rule out GBS. Specific antibody detection may have auxiliary diagnostic value for explaining atypical disease courses (13). These two cases together link a specific clinical phenotype to a particular immunological marker.

Moreover, there is a mismatch between the clinical presentation and EMG findings in our case. Clinically, his symptoms involve the right limbs, while EMG shows widespread abnormalities in all four extremities, including conduction block that meets electrodiagnostic criteria for demyelination (25). This phenomenon may result from distal functional compensation after early proximal nerve injury in GBS. As Berciano et al. noted, GBS pathology mainly affects proximal nerve segments, whereas distal electrophysiological abnormalities may arise from Wallerian degeneration secondary to proximal injuries (14). Thus, despite secondary electrophysiological alterations having occurred distally, motor function integrity may be relatively preserved by the compensation of residual neural function. Further, local factors such as variations in nerve antibody concentrations and differences in blood-nerve barrier integrity may further affect lesion distribution. Therefore, the asymmetry observed in our case may stem from the combined impact of these elements.

#### 3.2.2 Facial palsy

Both cases have the same right peripheral facial palsy. Our patient was initially diagnosed as idiopathic Bell’s palsy. However, the lack of response to glucocorticoids, along with subsequent development of limb weakness and sensory disturbances, cast doubt on the initial diagnosis. Up to 25-40% of peripheral facial paralysis cases have specific etiologies, and a poor response to standard treatment is a key indicator for misdiagnosis (15, 16). Heckmann et al. emphasize that GBS should be strongly suspected when motor dysfunction appears with facial palsy concurrently (17). Indeed, this patient also exhibited right limb weakness, consistent with GBS progression. A large retrospective cohort study by Fahimi et al. revealed that 10.9% of emergency department GBS cases were initially misdiagnosed as Bell’s palsy (18). This indicates that unilateral facial palsy is easily overlooked in early GBS diagnosis. Similarly, a multicenter case-control study during the Omicron pandemic found that among 41 patients diagnosed with GBS, 17 (41.5%) presented with cranial nerve involvement, including 13 (31.7%) with unilateral or bilateral facial paralysis (19). Together, these findings highlight the need to consider GBS when evaluating facial paralysis. Yet the mechanism of asymmetric facial paralysis in GBS remains unclear.

#### 3.2.3 Dyspnea

Unlike the case of Maharjan et al. (7), our patient developed acute dyspnea. Anti-GT1a antibody mediated dyspnea is primarily observed in GBS variants. Most scholars currently attribute dyspnea to antibody attacks on the glossopharyngeal and vagus nerves (6, 20), which cause severe bulbar palsy. However, given the absence of significant bulbar palsy, we hypothesized that his respiratory distress might stem from highly selective involvement of the diaphragm (C3-C5) and intercostal muscle nerve fibers by anti-GT1a Ab. The mechanism of this dyspnea without bulbar paralysis is unclear. Its precise pathogenesis requires further experimental validation. In addition, since the patient was unable to cooperate with pulmonary function tests, this study cannot accurately assess the degree of respiratory dysfunction. This is one of the main limitations of the study. Still, arterial blood gas analysis showed a PaO_2_ of 72 mmHg, a PaCO_2_ of 43 mmHg, and an SaO_2_ of 95%. At that time, the patient had not yet developed CO_2_ retention or required ventilatory support. Combined with clinical manifestations, these findings suggest early impaired respiratory function. Future studies should include this test to accurately assess the specific type and degree of pulmonary function impairment.

#### 3.2.4 Pain

The patient presented with right shoulder and back pain as his initial symptom. Similar to our case, the control case also has radicular pain at first. Biologically, Moulin et al.’s prospective study links early back pain in GBS to neuroinflammation, with the Lasegue sign exacerbating pain and suggesting radicular pain (21). Molecularly, Fewou et al. attribute this pain to radicular inflammation impairing endocytic clearance capacity (22). Dorsal root ganglion neurons may rapidly clear anti-ganglioside antibodies from cell membranes via cholesterol-dependent endocytic protective pathway. This process reduces complement mediated membrane attack complex formation and the subsequent cell lysis cascade, delaying the microstructural damage of neuronal cells (22). Notably, radicular pain is a common manifestation of GBS. Approximately two-thirds of GBS patients experience this symptom (21). It has no clear association with antibody subtypes. From a clinical perspective, this case shows radicular pain preceding motor symptoms, which may aid early suspicion of GBS.

### 3.3 Treatment and outcome

Following treatment with IVIG, the symptoms of this patient gradually improved. In typical GBS, recovery is relatively slower than in localized variants, and some patients are left with severe sequelae. One study reported that only 62% of patients achieved full recovery one year after onset, while approximately 20% died or had severe disability (23). By contrast, our patient recovered substantially within six months, showing a better outcome. Previous studies also indicate that GBS subtypes with focal onset typically have a favorable prognosis. A large-scale study showed a 98% recovery rate in paraplegic GBS patients within six months, versus 81% in quadriplegic patients (5). Similarly, most ophthalmoparalytic GBS patients achieve substantial functional recovery within weeks to months (6). The favorable outcome of this case may be attributed to unilateral confined immune attack and early IVIG therapy halting disease progression. Thus, for atypical GBS with unilateral onset, early recognition and timely immunotherapy may lead to good prognosis.

## 4 Conclusion

We report a rare GBS variant presenting with persistent unilateral limb weakness and facial palsy as the predominant symptoms, associated with anti-GT1a antibody positivity. Heightened awareness of such atypical variants is essential for prompt recognition and avoiding misdiagnosis. Larger case series are needed to confirm this phenotype in the future.

## Data Availability

The original contributions presented in the study are included in the article/supplementary material. Further inquiries can be directed to the corresponding author.
